# Wearable Technology and Machine Learning for Prediction of Performance-Based and Patient-Reported Outcome Measures: A Systematic Review

**DOI:** 10.3390/s26041218

**Published:** 2026-02-13

**Authors:** Eloise Milbourn, Jiaqi Lai, Dale L. Robinson, David C. Ackland, Peter Vee Sin Lee

**Affiliations:** 1 Department of Biomedical Engineering, The University of Melbourne, Melbourne 3052, Australia; eloise.milbourn@student.unimelb.edu.au (E.M.); jiaqi.lai@student.unimelb.edu.au (J.L.); dackland@unimelb.edu.au (D.C.A.); 2 School of Mechanical and Mining Engineering, University of Queensland, Brisbane 4072, Australia; dale.robinson@uq.edu.au

**Keywords:** wearable sensors, machine learning, PROMs, PBOMs, digital biomarkers, free-living monitoring, remote patient monitoring, digital health

## Abstract

Machine learning models informed by patient-generated wearable data can be used to predict patient-reported and performance-based outcome measures. This approach offers a promising alternative to traditional outcome monitoring, which is commonly limited by recall bias, discrete sampling, and healthcare resource constraints. The aims of this systematic review were to identify wearable-derived features strongly associated with performance-based and patient-reported outcome measures, to compare the predictive performance across machine learning approaches, and to consolidate methodological limitations and provide suggestions for future work. Following a systematic search of four databases (PubMed, Scopus, Embase, and IEEE Xplore), 18 eligible studies were identified, published between 2017 and 2024, spanning patients across eight disease categories. Most studies used wrist-worn devices measuring accelerometry, sometimes combined with heart rate, respiratory, or sleep metrics. Random forest and support vector machine models were the most common, while hidden Markov temporal models showed improved performance with access to longitudinal data. Predictive performance ranged from poor to excellent (AUC 0.56–0.92), and non-linear models generally outperformed linear models. Despite promising early results, most studies report similar limitations of small sample sizes, limited external validation, and difficulty achieving acceptable accuracy beyond binary predictions. Overall, these studies highlight the potential of wearable-informed machine learning for continuous and objective outcome assessment, but the consensus calls for further work to apply larger, more diverse longitudinal datasets and interpretable temporal modelling approaches to bridge the gap between the current proof-of-concept state and clinical translation.

## 1. Introduction

Patient-reported outcome measures (PROMs) and performance-based outcome measures (PBOMs) are essential tools for patient monitoring, enabling quantitative evaluation of disease state and intervention effectiveness for both healthcare providers and researchers [1,2]. PROMs may be generic and measure a patient’s overall quality of life, such as the Patient-Reported Outcomes Measurement Information System (PROMIS), or condition-specific, such as the MD Anderson Symptom Inventory. In contrast, PBOMs are physical tests designed to evaluate patient function, such as the Timed Up-and-Go (TUG) and 6-Minute Walk Test (6MWT). Both PROMs and PBOMs are crucial for capturing long-term quality of life, function, and symptom burden. These measures are imperative for monitoring diseases where factors like pain levels, quality of life, and functional status are affected, such as chronic diseases (arthritis, diabetes, cancer, chronic pain), and neurological conditions (Parkinson’s disease, stroke, traumatic brain injury) [3,4,5,6,7,8,9].

Despite their extensive use, PROMs and PBOMs are inherently limited. Questionnaires rely on patient recall and are susceptible to reporting bias, patient literacy levels, cognitive impairment, and respondent burden [10,11,12,13]. PBOMs also require face-to-face interaction, trained personnel, and clinical resources. Both the administration and the interpretation of PROMs and PBOMs require standardisation and, by their nature, allow data collection only at discrete time points, which may not capture fluctuations in patient condition [14]. These limitations can result in reduced accuracy and efficiency, which is exacerbated in environments with limited resources [15].

Integration of wearables such as smartwatches, fitness trackers, and wearable pendants into patient care has the potential to reduce reliance on traditional outcome measures, alleviate time and financial burdens associated with clinic visits, also cutting subjective reporting bias [16,17]. Devices provide real-time insights on metrics such as activity levels, vital signs, gait patterns, and sleep quality, through the collection of physiological variables like heart rate, respiratory rate, and skin temperature, and movement data like acceleration and step count. Beyond stand-alone measurement, wearables have the potential to improve the prediction of recovery trajectories and mobility levels, enabling the identification of patients with slower recovery, supporting early intervention [18,19]. Access to real-time patient metrics can increase the accessibility of outcome monitoring, enhance care, and simplify patient-clinician interactions [20,21].

Recent advances and widespread adoption of wearable technologies [22,23] have supported unobtrusive, continuous measurement of objective free-living patient data [14,24]. In turn, this has enabled device-assisted diagnosis and disease monitoring, including applications in cardiovascular disease [25], metabolic disorders [26], and neurological conditions such as Alzheimer’s and Parkinson’s disease [27,28,29]. Wearables can also monitor psychological states and detect adverse events such as falls [30].

Much of wearable integration in healthcare is due to the large, multimodal time-series datasets generated by devices. However, clinically meaningful interpretation of these datasets using traditional statistical approaches alone is limited. Machine learning proposes a powerful approach to analysing complex datasets and extracting meaningful patterns, enabling the prediction of patient outcomes [31,32]. Feature importance analysis is commonly used to quantify the relative contribution of individual wearable-derived features to model predictions, providing insight into which signals most strongly drive predicted outcomes. Machine learning methods have demonstrated utility in diagnostics [30], risk estimation [33], and outcome prediction [34].

**Figure 1:**
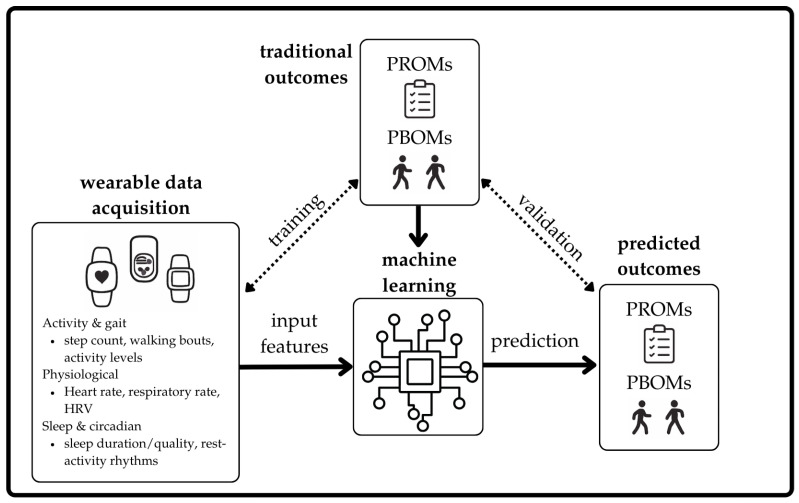
Wearable-informed machine learning for the prediction of patient-reported and performance-based outcome measures.

Applications of wearable informed machine learning to clinical outcome prediction (Figure 1) is beginning to appear in the literature with examples in cancer [35,36], heart disease [37], and surgery recovery, among others [38,39,40]. Gossec et al. (2019) used 3 months of step data collected from a wrist-based accelerometer and a Naïve Bayes Classifier to predict self-reported symptom flares in patients with rheumatoid arthritis or axial spondylarthritis [41]. The binary (flare/no flare) prediction model had a mean sensitivity of 96% and specificity of 97% over 1339 flare assessments [41]. More recently, Van den Eynde et al. (2023) monitored patients with Barth syndrome during a period of elamipretide treatment using a wearable (accelerometer, electrocardiography (ECG), and temperature sensor). Using features extracted from sensor data, an agglomerative hierarchical clustering model grouped patients based on four PBOMs (6MWT, balance, handgrip, 5 x sit-to-stand time) and two PROMs (PROMIS fatigue score and Barth syndrome symptom assessment). Model accuracies ranged from 60% to 93% when categorising top versus bottom performers across three clinic timepoints, and 100% across all outcomes when predicting responders to treatment versus non-responders. Key predictive features included nighttime minimum and maximum heart rate, daytime maximum heart rate, and daytime respiratory rate [42]. These examples demonstrate the emerging feasibility of leveraging wearable-derived features and machine learning models to predict PROMs and PBOMs across diverse disease contexts.

Despite these encouraging findings, many limitations of PROM and PBOM prediction from wearable data-informed machine learning remain, and results of preliminary studies employing these methods should be interpreted with caution. Most studies report similar limitations. This includes small sample sizes, especially with respect to machine learning, that can easily result in overtraining of models, exaggerating accuracy and limiting generalisability, and incomplete disease coverage adds to the lack of generalisability when it comes to wider applications of such techniques [41,42,43,44].

In complex machine and deep learning models where predictions arise from high-dimensional, nonlinear parameter interactions, there exists a trade-off between accuracy and interpretability. Accurate predictions of patient outcomes may be possible but will lack external validity in clinical settings if the underlying physiological mechanisms driving patient conditions remain unknown [41,45]. Patient compliance with wearable sensors is unreliable and varies within and between studies [36,37,46,47,48]. With the aim of such methods being to capture continuous activity and biomarker data, even short periods of non-wear lead to gaps in datasets, which can be difficult to normalise and account for. Additionally, not all studies report compliance rates or methods used to account for missing data, and varying techniques exist among those that do. Finally, model selection depends on data type, sample size, feature set, labelling, and target outcomes, and there is currently no consensus on optimal machine learning approaches for outcome monitoring, reflecting the exploratory stage of research in this field [35,45].

This review aims to synthesise published studies that predict PROMs and PBOMs using wearable device data and machine learning. We aim to (i) identify wearable-derived features strongly associated with PROMs and PBOMs, (ii) compare predictive performance across machine learning approaches, and (iii) highlight methodological gaps and opportunities for integrating wearable-based outcome prediction into clinical practice. By consolidating current evidence, this review seeks to inform future work, accelerating the translation of wearable and machine learning technologies from a research concept to a real-world outcome assessment tool.

## 2. Materials and Methods

The review protocol was registered with the International Prospective Register of Systematic Reviews (PROSPERO), registration number CRD420250567500, and conducted following the Preferred Reporting Items for Systematic Reviews and Meta-Analyses (PRISMA) statement [49].

### 2.1. Search Strategy

Studies were identified by a systematic search performed by the reviewers of the following databases: PubMed. Scopus, Embase, and IEEE Xplore. The search covered all studies published between 2010 and 12 July 2024 (the date of search) containing the search terms, or variations of “wearables” AND “prediction” AND “outcomes” (Table 1).

### 2.2. Eligibility Criteria

Eligibility criteria included empirical research studies available in English where free-living patient data was collected by one or more non-invasive wearable devices and analysed with machine learning, with the aim to predict or classify patient-reported or performance-based outcomes directly.

Studies were excluded if the patient-reported outcomes were not generated by the patient or if data were not collected during free-living (e.g., hospital stay). Additional exclusion criteria included invasive devices that required penetration of the skin barrier (e.g., implants and continuous glucose monitors), studies of psychiatric disorders, and studies that lacked detailed, relevant information required for comparison (e.g., type of wearable, machine learning model used, qualitative results).

### 2.3. Study Selection

Records identified in the search were imported into Covidence software (Veritas Health Innovation Ltd., Melbourne, Australia), which automatically removed the majority of duplicates. Remaining duplicates were then manually removed before two reviewers independently screened all titles and abstracts for relevance. Conflicts were resolved by discussion between the reviewers. Both reviewers then screened the full texts of the remaining studies, including or excluding papers based on the pre-defined inclusion and exclusion criteria. Conflicts were again resolved by discussion between the reviewers.

### 2.4. Data Extraction

Data was extracted independently by two reviewers using a jointly made custom template. Data extracted were summaries of study design, patient information, and sample size, inclusion and exclusion criteria, wearable type, location, and duration, outcome type and frequency, machine learning model(s) and input features, feature analysis, results observed, and study limitations. Extracted data was compared between reviewers, and conflicts were resolved by discussion. Model performance metrics reported in studies varied by model category (e.g., classification vs. regression) and outcome format (e.g., binary classification vs. multiclass classification). For each model category, the following metrics were extracted and prioritised in analysis, where available: deep learning models; accuracy (ACC) (mean absolute error where ACC not reported), sequence models; Area Under Curve (AUC) and Pearson’s correlation coefficient for binary and multiclass outcomes, respectively, clustering; F statistic (ACC where F statistic not reported), classification; ACC (Cohen’s kappa where ACC not reported), regression AUC and Spearman’s correlation coefficient for binary and continuous outcomes, respectively (Goodman–Kruskal gamma where AUC not reported). F1 scores were also extracted and used in analysis, where they allowed for further comparison between model performance.

### 2.5. Quality Appraisal

The quality of each included study was evaluated by two reviewers using a custom quality assessment checklist devised to evaluate key study characteristics within the scope of the review. The evaluation tool, comprising 11 questions, was derived from the Downs and Black checklist [50], the STROBE statement [51], and established reliability and feasibility appraisal tools [52]. Each question returned a score of 0, 1, or 2, depending on whether the content was not addressed, partially addressed, or clearly addressed, respectively. Scores from the 11 questions per paper were summed, and studies were classified as being of high quality (≥20), moderate quality (<20 and ≥15), or low quality (<15). Each study was scored individually by two reviewers, and conflicts were resolved by discussion. Questions of the quality assessment tool:

1. Are the research aims or objectives clearly described?
2. Are the patient inclusion and exclusion criteria clearly stated?
3. Are the cohort characteristics clearly described (sample size, male/female, disease state)
4. Is the wearable device, location, and monitoring period clearly described?
5. Are the patient-reported outcomes or functional tests clearly described?
6. Are the machine learning techniques clearly described?
7. Are the model input features clearly stated?
8. Is the method used to address missing data clearly described?
9. Are the main outcomes and key findings clearly stated?
10. Are study limitations and biases discussed?
11. Does the discussion include generalisability (external validity) of the study results?

## 3. Results

### 3.1. Search Results and Quality

**Figure 2:**
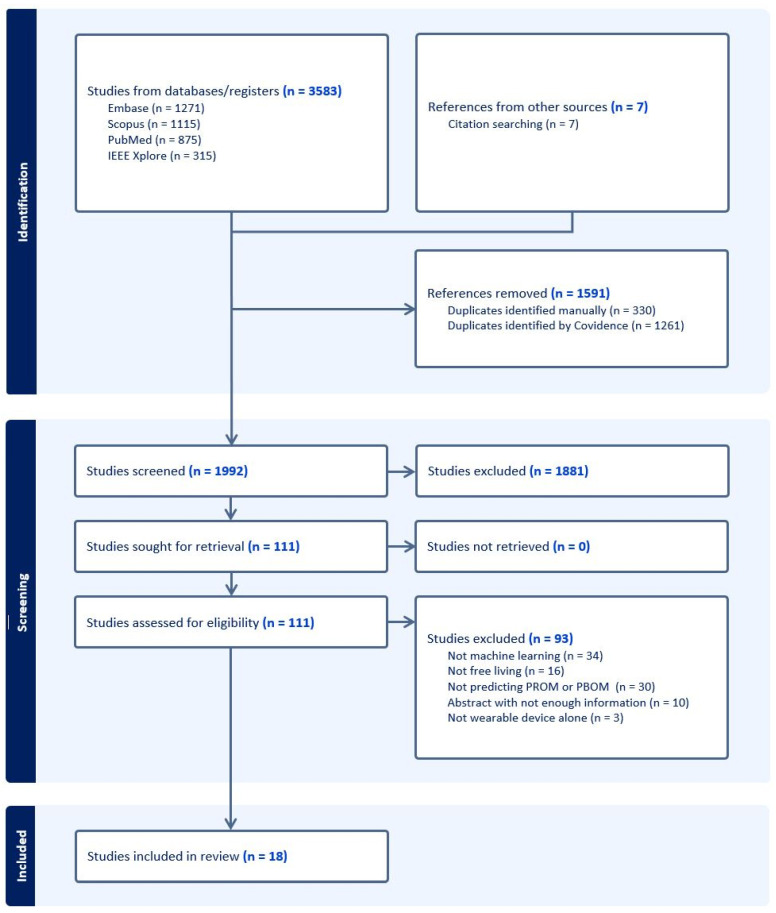
PRISMA flow diagram exported from Covidence software. Abbreviations used: PROM, patient-reported outcome measure; PROM, performance-based outcome measure.

The search returned a total of 3576 records plus seven from manual or citation searching. After removal of 1591 duplicates and screening, 18 studies met the full criteria (Figure 2).

The main reasons for the exclusion of studies were not using machine learning as the primary analysis method, not predicting patient-reported or functional test outcomes, and wearable data not collected during free-living.

**Figure 3:**
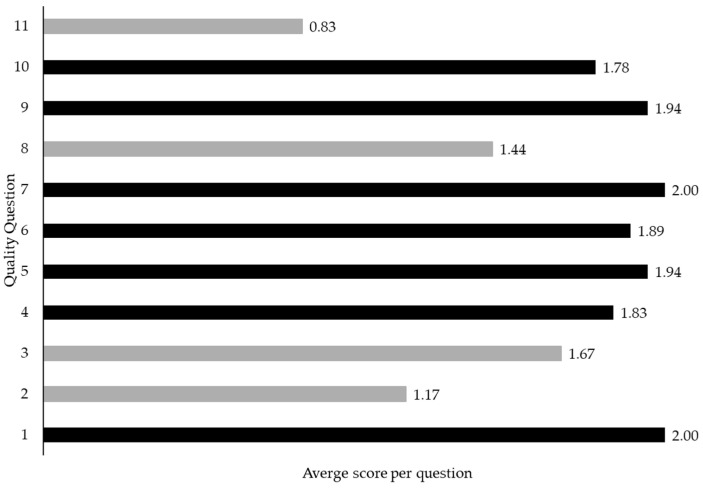
Average score per question of the quality assessment tool. Questions shown in grey received below the average score of 1.68.

Of the 18 studies included in the review, nine (50%) were classified as high quality, seven (39%) as moderate quality, and two (11%) as low quality. Quality scores ranged from 12 to 22, with a mean of 18.5. Studies scored highest on questions relating to aims and objectives, clearly stating model input features, and describing main outcomes and key findings. Overall, four questions received below the average score of 1.68. These questions were related to reporting of patient inclusion and exclusion criteria, patient characteristics, handling of missing data, and comparisons with standard outcome monitoring (Figure 3).

### 3.2. Characteristics

The included studies were published between 2017 and 2024. Most studies (61%) were published from 2023 onwards. Sample sizes used to build prediction models ranged from six [44] to 2001 [53] with a mean sample size of 188. All studies were observational in nature, except for one randomised controlled trial [42]. Free-living wearable monitoring periods ranged from 6 h [35] to 6 months [48,54], with a mean of 54 days.

Twelve studies [36,39,40,41,42,43,45,46,47,53,54,55] reported the mean age of a total of 3231 patients; of these, the mean age was 63.2 years. Sex distribution was reported in eleven studies [36,39,40,41,42,43,45,46,47,48,55], and of the total 3216 patients, 60% were female.

**Figure 4:**
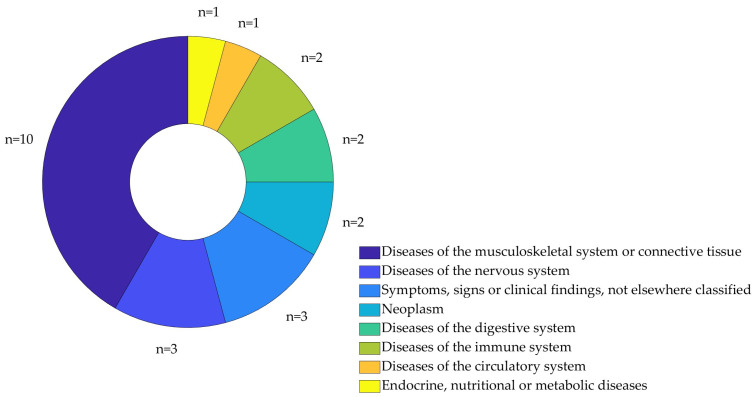
Diseases covered are categorised according to the World Health Organisation International Classification of Diseases—11.

Patients monitored in the 18 studies spanned eight disease classifications according to the World Health Organisation International Classification of Diseases—11 (ICD-11) [56] (Figure 4). Two papers [45,55] monitored patients ranging over multiple ICD-11 categories.

### 3.3. Wearable Devices and Placement

Across the reviewed studies, a variety of wearable devices and sensor types were employed to collect objective physiology and activity data, with placement locations varying (Table 2). Wrist-worn devices were most prominent (*n* = 11), followed by the waist and chest (*n* = 2 each) (Table 2). Seventeen different devices were identified, and two were not specified. Only one study used multiple devices on the same patient [46].

Fitbits were most common (six studies), typically combining accelerometers and Photoplethysmography (PPG). Most wearable devices house an array of sensors, most frequently including accelerometers (all studies), PPG (*n* = 9), barometers (*n* = 8), gyroscopes (*n* = 4), and temperature sensors (*n* = 4). Reported values of patient adherence to wearable devices ranged from 59% [46] to 85% [48,54].

### 3.4. Machine Learning

**Figure 5:**
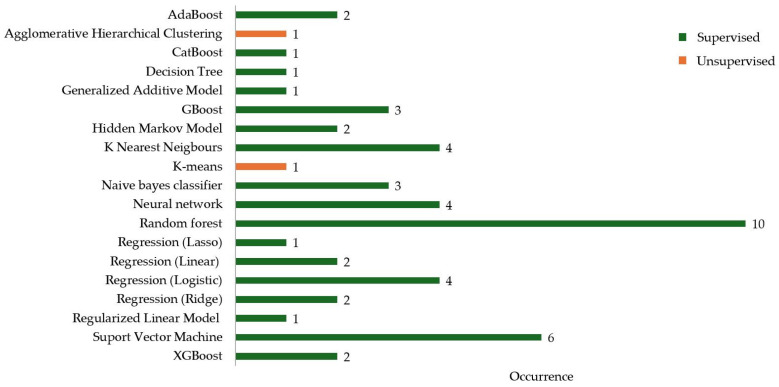
Occurrence of Machine Learning models within studies.

Across the included 18 papers, 19 different machine learning methods were reported, of which 17 were supervised and two unsupervised (Figure 5). Of the supervised model types, nine were classification, six regressions, one sequence, and one deep learning. Both of the unsupervised models were clustering models. Random forest (*n* = 10) and Support Vector Machine (*n* = 6) were the most frequent.

Studies varied in how they classified patients based on outcomes. Externally anchored classification models grouped patients via discrete questionnaire responses [35,38,41,43,44,45,48,54,55,57] or reported outcomes above/below symptom severity according to t-scores [37,47,48]. Within-cohort approaches classified patients as above/below the cohort mean for a given outcome [36,42,45], stratifying by interquartile ranges within the cohort [53], or by highest/middle/lowest scores within cohorts [46]. Change-based classification grouped patients based on individual outcome changes over time, such as responders/non-responders to treatment [42], and individuals who did/did not achieve clinically meaningful improvements [40]. Notably, one study attempted to predict timed-up-and-go performance as a continuous outcome [39]. Of the 18 included studies, the majority (*n* = 16) evaluated models using internal validation approaches [36,37,38,40,41,42,43,44,45,46,47,48,53,54,55,57], utilising the same overall recruitment pool for both training and testing, typically splitting the data to ensure the model was evaluated on “unseen” samples from the same population. The remaining two studies [35,39] performed external validation strategies, testing models on a completely independent dataset that was not used during any part of the training or parameter selection phase.

Nine studies [35,37,40,43,44,45,47,48,57] assessed the performance of multiple machine learning models on the same datasets (Table 3). The mean paper quality score was 18.0, with a range from 12 to 21. Two studies were of low quality [35,44], three of moderate [37,43,57] quality, and four of high quality [40,45,47,48]. Due to high heterogeneity in study populations, prediction methods, and outcomes, a meta-analysis of the field was not feasible; a more narrative synthesis is presented. As between-study comparisons are limited by the heterogeneous nature of both the populations and approaches, the analysis of machine learning methods will focus primarily on studies that report performance across multiple models on the same dataset.

Bahej et al. (2018) predicted Short Form 36 (self-reported measure of health) questionnaire scores in patients with chronic and degenerating musculoskeletal conditions, classifying patients based on their answers to questions related to walking and activities of daily living. A binary random forest model performed best, achieving up to 82% accuracy, compared to a support vector machine model, which achieved up to 76% accuracy [43]. In both regression and classification problems, Pantelidou et al. (2023) also found random forest models to outperform support vector machine models as well as 8 other algorithms, reporting accuracy of up to 96% when predicting fatigue from wearable data in a healthy population, before applying this model to a subset of cancer patient data [35]. Similarly, Patterson et al. (2023) [48] compared logistic regression, support vector machine, K Nearest Neighbors, random forest, and CatBoost performance in predicting 12 quality-of-life domains and disease-specific questionnaire outcomes in chronic pain patients. Averaged across all outcomes, again, random forest achieved the highest accuracy, reporting 77% [48]. Hinchliffe et al. (2024) compared the performance of support vector machine, K Nearest Neighbors, random forest, and Naïve Bayes classifiers to detect physical and mental fatigue in neurodegenerative or immune disorders. All models performed poorly, but the random forest reported the best accuracy for intersubject cross-validation (53%) and the support vector machine for intrasubject cross-validation (56%) [45].

In contrast, Meng et al. (2019) and Rao et al. (2023) investigated the performance of independent per-week random forest models against temporal hidden Markov models (incorporating correlations of PROM score states across weeks) when predicting PROMIS domains in heart disease [37] and rheumatoid arthritis [47]. In both studies, the hidden Markov model significantly outperformed the random forest model (*p* < 0.05).

Sarwar et al. (2022) compared seven machine learning models for the prediction of three PROMIS domain scores reported by chronic pain patients using accelerometer data. The best performing models were logistic regression and gradient boosting, achieving Area Under Curves (AUC) of 0.67 and 0.56, respectively [57]. Also finding regression to outperform other models, Greenberg et al. (2024) studied patients’ recovery from lumbar spine surgery, predicting results of PROMIS pain interference and Quality of Recovery-15 questionnaire scores. Of the six models tested, logistic and ridge regression achieved the best AUCs of 0.69 (Quality of Recovery-15) and 0.62 (PROMIS pain interference), respectively [40]. Finally, Pope et al. (2023) predicted Parkinson’s patients’ self-reported tremor scores using polynomial regression and a shallow neural network, with the neural network performing best in five of six patients [44].

### 3.5. Predicting Quality of Life

Quality of life questionnaires are measures for quantifying a patient’s overall health in longitudinal monitoring of chronic disease, as well as following treatment interventions. Three quality-of-life tools (PROMIS, Short Form-36 questionnaire, and Veterans RAND 12-item Health Survey) were identified across eight papers and included in the review. Quality scores ranged from 16 to 21, with a mean of 19 (Table 4).

PROMIS Physical function was predicted from wearable data in four studies [37,47,48,57], with fair results for binary predictions: AUC 0.79 (heart disease) [37], 0.78 (chronic pain) [48], and 0.75 (rheumatoid arthritis) [47]. In contrast, a 3-class classification model in chronic pain patients performed poorly (AUC 0.56) [57]. Feature importance analysis revealed the key features in physical function prediction were total steps, total distance/activity, time, or distance travelled during light activity [37,47], min and max heart rate [47] and intradaily variability (a measure of circadian disturbance where intradaily variability elevation indicates more daytime napping or nighttime arousal) [57]. PROMIS fatigue was predicted in four studies [37,42,47,48], with results of binary prediction models ranging from poor AUC 0.65 [37], through fair AUC 0.70 [47], to good AUC 0.89 [48], accuracy 87% [42]. Feature importance analysis revealed the most important features in fatigue prediction to be steps, basal metabolic rate, calories, and total distance [37] and the max and min heart rate during the day and night [42]. PROMIS sleep disturbance was predicted in four studies [37,47,48] using binary prediction models, ranging in performance from poor AUC 0.66 [37] and 0.65 [47] to good AUC 0.88 [48]. Important features in sleep disturbance prediction were steps, basal metabolic rate, calories, and total distance [37].

PROMIS anxiety and depression were predicted in two studies, with binary prediction models reporting AUCs of 0.61 (anxiety) and 0.59 (depression) [37], and AUCs of 0.92 (anxiety) and 0.89 (depression) [48]. Feature analysis by Meng et al. (2019) reveals similar features of most importance in both anxiety and depression models, including calories and resting heart rate (anxiety and depression), steps (depression), and total distance walked (anxiety) [37]. PROMIS social roles scores were predicted in two studies [47,48], with binary prediction model performance ranging from poor AUC 0.67 [47] to good AUC 0.88 [48]. Neither study reported on the important features of social roles.

Bahej et al. (2018) achieved 73% overall accuracy when using a random forest model to classify patients with chronic and degenerating musculoskeletal disorders based on their response to five mobility and activities of daily living questions from the Short Form-36 questionnaire. The most important predictive features were reported as the standard deviation of gait speed, walked distance, activity count features, and number of steps [43]. Similarly important features relating to overall activity levels underpinned the clustering of total joint arthroscopy patients based on their six-week post-surgery Veterans RAND 12-Item Health Survey physical component scores. Bini et al. (2019) reported the model with the largest f-statistic (17.37) used only personal activity intelligence (an individualised cumulative weekly score reflecting the amount and intensity of physical activity considering sex, age, resting, and maximal heart rate) [46].

### 3.6. Predicting Disease-Specific Outcomes

Eleven studies were identified as predicting one or more disease-specific outcomes, with quality scores ranging from 12 to 22 and a mean of 18.3 (Table 5).

#### 3.6.1. Neurodegenerative and Immune-Mediated Inflammatory Diseases

Four studies predicted self-reported outcomes across patients with Parkinson’s disease, Huntington’s disease, rheumatoid arthritis, systemic lupus erythematosus, primary Sjögren’s syndrome, axial spondyloarthritis, and inflammatory bowel disease [41,44,45,55]. Antikainen et al. (2022) used respiratory rate data from a range of patients with neurodegenerative and immune-mediated inflammatory diseases to predict binary Karolinska sleepiness scale scores (sleepy/non-sleepy states), reporting 63% accuracy [55]. In a similar study using only gait characteristics data from this cohort, a binary (above/below cohort mean) prediction model of self-reported physical and mental fatigue returned accuracies of 57% and 56%, respectively [45]. Hinchliffe et al. (2024) reported that the most important features were bout length variability (irregularity in activity/rest cycles), acceleration vector magnitude (intensity of movement), step length asymmetry, and gait rhythm. Overall activity volume was less important, suggesting fatigue may not be reflected by a reduction in walking volume, but by subtly altered movement patterns seen in increased variability of gait, balance, and rhythm [45].

Gossec et al. (2019) grouped rheumatoid arthritis and axial spondyloarthritis patients via a binary (self-reported symptom flare/no flare) classifier with a mean positive predictive value of 91% and a negative predictive value of 99%. Post hoc analysis highlighted that deviations in regular activity patterns (steps/minute) at certain points of the week (in particular Saturday afternoons) had the strongest associations with flare detection [41]. Finally, when predicting self-reported tremor intensity (none/slight or mild/moderate or bothersome/very bothersome or severe) in Parkinson’s patients, Pope et al. (2023) reported a mean absolute error ranging from approximately 0.30 to 0.65. The most important acceleration features were the power and stability of oscillations in the tremor frequency range (4–7 Hz) [44].

#### 3.6.2. Musculoskeletal Conditions

Post-surgical outcomes were predicted in patients recovering from lumbar spine [40] and knee [38] surgery. Using pre-operative wearable data, Greenberg et al. (2024) predicted binary 30-day post-operative Quality of Recovery-15 questionnaire score above/below 122 (threshold for good or excellent recovery) with an AUC 0.69. Sleep characteristics, greater activity amount and intensity, and presurgical activity trend were predictive of improved recovery [40]. With the addition of more in-depth physiological metrics, Wibowo et al. (2023) achieved a higher AUC of 0.86 using 25 days of post-knee surgery data to classify patients’ self-reported pain (no or mild pain/moderate or severe pain), using actigraphy, blood pulse wave, heart rate, oxygen saturation, perfusion index, respiratory rate and skin temperature as input features [38].

#### 3.6.3. Chronic Pain

Heros et al. (2023) and Patterson et al. (2023) followed a cohort of chronic pain patients for six months post implantation of spinal cord stimulation systems and classified patients based on PROM scores using wearable data. Patterson et al. (2023) reported accuracies of 70%, 77%, 83%, 84%, and 85% for predictions of the Oswestry disability index, Numerical Rating Scale, Patient Global Impression of Change, Pain Catastrophising Scale, and Patient Health Questionnaire-9, respectively [48]. Using a similar approach, but also featuring Principal Component Analysis, Heros et al. (2023) reported accuracies of 78% (Numerical Rating Scale) and 75% (Patient Global Impression of Change) [54]. Most important features were reported for Numerical Rating Scale prediction, including exercise frequency, step count features, and heart rate variability [48,54], heart rate stand time, and days post-implantation [48].

#### 3.6.4. Cancer

Two studies assessed symptom prediction in oncology populations. Low et al. predicted daily symptom burden (high/average/low) as reported by the MD Anderson Symptom Inventory during chemotherapy using sleep and activity features. The model achieved 78% accuracy, with number of steps and minutes lightly active as the most important features. The authors also reported that using only activity tracking features from patients’ phones (most common activity, standard deviation of accelerometer, and minimum acceleration) provided even higher accuracy of 89% [36]. Pantelidou et al. (2023) applied a binary model to predict self-reported fatigue (fatigue/no fatigue) using data from control participants, which achieved an accuracy of 68% when applied to cancer patients, whilst restricted to overlapping wearable features between datasets (heart rate and skin temperature) [35].

#### 3.6.5. Barth Syndrome

One study investigated disease-specific fatigue outcomes in Barth syndrome [42]. Patients were classified by self-reported fatigue status (above/below cohort median) and by response of reported fatigue to treatment (responders/non-responders). The model showed limited ability to discriminate between most and least fatigued (60% accuracy), whilst responder/non-responder discrimination achieved 100% accuracy from pre and post treatment wearable data alone. Key predictive features included daytime minimum, mean, and maximum heart rate and daytime respiratory rate [42].

### 3.7. Predicting Performance-Based Outcomes

Performance-based outcomes are physical tests designed to measure the level of physical function of a patient. This review details three studies that aimed to predict one or more performance-based outcomes. All studies were of high quality with scores ranging from 20 to 22 and a mean of 20 (Table 6).

Saporito et al. (2019) estimated TUG performance from free-living wearable data and tested model sensitivity by predicting TUG performance of 15 patients recovering from total hip arthroplasty. A regularised linear model achieved a strong correlation (ρ 0.67) with standardised TUG times, demonstrating high test–retest reliability (Intraclass Correlation Coefficient 0.94) [39].

Agarwal et al. (2018) predicted the 400 m walk test, 20 m walk test, and five times sit-stand test from free-living accelerometry in knee osteoarthritis patients. “Function profiles” were defined based on average daily minutes spent in various acceleration “pattern classes”. Multi-class classification models grouped subjects into three classes based on cohort performance (lowest/highest/interquartile range). Generalised Additive Models reported Goodman-Kruskal Gamma statistics of 0.62, 0.53, and 0.51 for the 400 m walk test, 20 m walk test, and five times sit-stand test, respectively [53]. Function profiles, along with body mass index (BMI), age, sex, height, and osteoarthritis subcohort status, were inputted to Generalised Additive Models, including the activity profiles in the models, in addition to demographic and clinical data, which improved the Gamma statistic by 4–10% [53].

Using metrics from a torso-worn wearable device, Van den Eynde et al. (2023) classified patients based on PBOM scores using two methods: above/below cohort mean score, and responders/non-responders to drug treatment. Agglomerative Hierarchical Clustering models demonstrated accuracies of 93%, 80%, 73%, and 60% for 6MWT, balance score, five times sit-stand test, and handgrip strength, respectively. These models distinguished between responders and non-responders with 100% accuracy for all outcomes [42].

The most accurate version of Saporito et al.’s (2019) TUG model relied on features derived from walking and sit-to-stand transitions, including walking quantity and quality, chair rise quality, and active and inactive bout durations [39]. Agarwal et al. (2018) reported movement pattern classes associated with higher daily mean activity counts and fluctuating levels of exertion correlated with superior walking and sit-to-stand performance [53]. In Van de Eynde et al. (2023), the most discriminative features for clustering patients based on predicted performance and treatment response were derived from min and max heart rate and respiratory rate values during the day and night [42].

### 3.8. Predicting Mobility Outcomes

Eight studies predicted mobility outcomes from wearable data, either as self-reported questionnaires [37,43,47,48,57] or performance-based tests [39,42,53]. Study quality ranged from 16 to 22, with a mean of 19.6 (Table 7).

Four studies predicted PROMIS physical function [37,47,48,57]. In patients with stable ischemic heart disease monitored over 12 weeks, a Hidden Markov Model achieved a mean AUC of 0.79 for binary classification (above/below symptom severity). Rao et al. (2023) also used a Hidden Markov Model, but in patients with rheumatoid arthritis. Binary classification (normal vs. moderate/severe severity) achieved an AUC score of 0.75 [47]. When predicting PROMIS physical function scores (above/below symptom severity) in chronic pain patients, Patterson et al. (2023) reported a similar AUC of 0.78 [48]. Also, in chronic pain patients, Sarwar et al. (2022) used features derived from wrist-worn actigraphy to classify patients based on PROMIS physical function, achieving an average AUC of 0.56 [57]. Feature analysis revealed the total steps and activity [37,47,57], lightly active volume [37], minimum heart rate [47], sleep, and Rest-Activity Circadian Rhythm [57] to play significant roles in PROMIS physical function prediction.

Bahej et al. (2018) used data from a waist-worn accelerometer to predict scores from a subset of Short Form-36 questions in patients with chronic and degenerating musculoskeletal conditions. Binary classifiers predicted scores related to walking difficulty and activities of daily living limitations from 162 acceleration features, achieving a mean accuracy of 73%, with the most predictive features being the standard deviation of speed, walked distance, activity counts, and number of steps [43].

The outcomes of four walking mobility tests were predicted: TUG [39], 400 m walk test [53], 6MWT [42], and 20 m walk test [53]. Saporito et al. (2019) predicted TUG time in seconds and reported a correlation (*ρ =* 0.67) between actual and predicted times. The model also demonstrated good discriminatory power (AUC 0.89) to identify subjects with TUG > 10 s (the cutoff value for frailty) as well as sensitivity to mobility changes, tracking significant reductions in TUG time as mobility improved between weeks two and six post total hip arthroplasty surgery [39]. Agarwal et al. (2018) classified patients based on predicted 400 m walk test and 20 m walk test performance, reporting a classification agreement of γ 0.62 (Goodman and Kruskal’s Gamma) for the 400 m walk test and a lower value (γ 0.53) for the 20 m walk test [53]. Van den Eynde et al. (2023) clustered top and bottom performers based on median cohort predicted 6MWT times, achieving an accuracy of 93% [42]. Feature importance analysis revealed that walking quantity, walking quality, chair rise quality, and active and inactive bout durations were the most important for TUG predictions [39]. Steady walking bouts of moderate intensity predict endurance (400 m walk test) and shorter, variable activities (stand–walk transitions, interrupted walking) predict walking speed (20 m walk test) while both outcomes show a consistent benefit from short-to-moderate exposure (≤20–25 min/day) in moderate–vigorous classes. Still, too much high-intensity fluctuating activity may harm function in some cases [53]. Finally, respiratory rate during night and day, and minimum and maximum nighttime heart rate were the most important features for 6MWT prediction [42]. Interestingly, the only study that included physiological variables, in addition to activity, found heart rate and respiratory rate to be more important in the prediction of sustained walking [42].

Agarwal et al. (2018) and Van den Eynde et al. (2023) both predicted five times the sit-stand test times from wearable data, with results of γ = 0.51 [53] and accuracy = 73% [42], respectively. Both models showed inferior performance compared to the longer walking tests. Feature analysis revealed the most predictive features to be: minimum heart rate (daytime and nighttime), and daytime maximum heart rate and respiratory rate [42], as well as interrupted moderate activity (short bouts with variability reflecting sit/stand transitions) and some moderate-to-vigorous activity [53].

Finally, Van den Eynde et al.’s (2023) model also predicted performance (above/below cohort mean) on handgrip strength and balance tests, with 60% and 80% accuracy, respectively [42]. The most predictive features were maximum and minimum daytime and nighttime heart rate (handgrip), daytime respiratory rate, and minimum and maximum nighttime heart rate [42].

### 3.9. Predicting Pain

Six papers [38,40,47,48,54,57] included a prediction of one or more outcome measures directly related to the prediction of pain. Quality scores ranged from 15 to 22, with a mean of 19.7 (Table 8).

Four studies aimed to predict PROMIS pain interference scores [40,47,48,57]; one on PROMIS pain intensity scores [57], and three on the Numerical Rating Scale [38,48,54], across patient groups with chronic pain [48,54,57], rheumatoid arthritis [47], and those recovering from lumbar spine [40] and knee [38] surgery.

In chronic pain patients, multi-class classification models have been used to group patients into mild/moderate/severe pain categories as reported by questionnaires from five days [57] and six months [48,54] of wearable data. The F1 scores of each study with the highest performing classification models were 0.60 [57], 0.73 [54] and 0.77 [48].

One study used pre-operative wearable data to predict whether patients recovering from lumbar spine surgery would have a clinically meaningful reduction in reported pain at post-operative day 30, with an AUC of 0.62 [40]. Wibowo et al. (2023) classified patients recovering from knee surgery into binary (no or mild/moderate or severe) and 4-class (no/mild/moderate/severe) categories of self-reported pain following 25 days of post-operative monitoring, with a classification accuracy of 86% and 90% [38]. Rao et al. (2023) categorised PROMIS pain interference scores (normal vs. severe pain) in patients with rheumatoid arthritis, achieving an AUC of 0.66 [47].

Using Shapley Additive exPlanations (SHAP) analysis, temporal features such as the variability of daily heart rate [40] and Rest-Activity Circadian Rhythm features, particularly Intradaily Variability [57] were shown to be the most predictive features of PROMIS pain scores, with elevated variability reflecting increased reported pain. Other important features reported were the minimum heart rate [48,54], heart rate variability, pain history, step count, and stand time [54] and the number of days pre/post intervention [48].

## 4. Discussion

This review identified 18 studies that applied wearable-derived data and machine learning to predict patient-reported and performance-based outcomes across eight ICD-11 [56] disease categories. The most prominent types of devices were wrist-worn, specifically Fitbits (six studies). The most common sensors housed by the wearables were accelerometers (all studies), PPG (nine studies), and barometers (eight studies). Predictive performance varied from poor to excellent, with AUC values ranging from 0.56 to 0.92. Random Forest and Support Vector Machine models were the most common approaches, while studies that incorporated temporal Hidden Markov Models to account for longitudinal wearable data and outcome trends demonstrated improved performance over independent, cross-sectional models [37,42,47]. Of the nine included studies that directly compared the performance of linear vs. non-linear models, seven reported that the best-performing model was non-linear (particularly ensemble methods like Random Forest) [35,37,43,44,45,47,48]. Non-linear models may have superior abilities to handle the complexity of relationships between often noisy and high-dimensional wearable data and PROMs and PBOMs. However, these performance gains were often modest and task-specific, with linear models remaining competitive, especially in studies using extensive feature engineering or with limited sample sizes. Linear models, where high in predictive accuracy, remain valuable for clinical utility, allowing researchers and clinicians to interpret which features drive medical prediction. Mobility, sleep disturbance, and pain interference were generally the most successfully predicted outcomes, while fatigue-related outcomes showed more variable results. Overall, PROM prediction models achieved moderate accuracy in some studies (e.g., AUC of 0.76 for physical function [37]), but performance was generally lower where subjective fatigue scores were used [35,37,42,45,47]. Within studies, binary classification models outperformed multiclass classification and continuous variable approaches, a pattern that may be exaggerated by small sample sizes and class imbalances [38,39,47].

A subset of studies used post hoc feature importance analyses (e.g., SHAP) to enhance interpretability [35,37,40,41,43,45,47,48,54,57], a critical step towards clinical utility through the identification of wearable factors most strongly influencing outcome predictions [58,59]. Predictive features were typically statistical features of physiologically interpretable variables such as step count, heart rate, respiratory rate, sleep duration, and day-to-day variability, demonstrating that clinically relevant information can be extracted from unobtrusive, off-the-shelf wearables.

Employing machine learning approaches for the interpretation of wearable sensor data offers a means to assess, continuously and objectively, disease progression, patient status, and treatment response with substantial clinical implications [60,61]. Rather than relying solely on current monitoring methods, several studies demonstrate that outcomes can be predicted before intervention or recovery milestones. For example, Greenberg et al. (2024) predicted binary (above or below the threshold for good/excellent recovery) 30-day post-operative Quality of Recovery-15 questionnaire scores from presurgical sleep characteristics and activity [40]. These approaches extend beyond passive tracking to genuine prognostic capability. Such predictive models could support remote monitoring, early detection of clinical deterioration, personalised rehabilitation planning, and risk stratification for targeted therapy selection [62,63].

Prediction of PBOMs such as the TUG or 6MWT from wearable data could enable remote functional assessment, reduce hospital visits, and allow continuous post-operative or rehabilitative monitoring. Additionally, continuous measures may capture long-term temporal trends more effectively than isolated clinic assessments, providing richer insights into how symptoms affect day-to-day functioning [40,55]. Continuous detection of worsening physical or psychological symptoms could also enable “just-in-time” adaptive interventions: for example, when symptom levels rise, an automated alert or self-management prompt could be delivered, supporting timely intervention and improved quality of life [64,65]. Saporito et al. (2019) demonstrated the sensitivity of their prediction model to changes in patients’ mobility following total hip arthroplasty surgery, as reflected in remote TUG prediction. Interestingly, this model was trained on only three days of quantitative characterisation of activities of daily living data collected by a wearable in a healthy population [39]. This result shows that remote mobility prediction in clinical groups may be possible from models trained on data from healthy populations, a finding of particular interest in a machine learning context where large datasets are required for appropriately fitted models.

Mobility is a recognised indicator of disease progression and all-cause mortality [29,66,67], and remote measurement of mobility is highly valuable for continually assessing frailty and recovery [68,69,70,71]. Step count and activity duration were repeatedly reported as dominant predictors of patient-reported mobility [37,43,47,48], while Agarwal et al. (2018) found that short bouts of moderate activity interspersed throughout the day, rather than prolonged sedentary periods or intense exercise, were the strongest wearable correlates of lower-extremity function [53]. Studies predicting wearable-derived PBOMs further confirmed the feasibility of functional prediction, reporting strong correlations between predicted and real TUG (ρ = 0.70; AUC 0.89) [39], and achieved up to 93% classification accuracy for 6MWT status using clustering approaches [42]. Collectively, these results show that both simple activity metrics (steps, distance, duration) and more complex composite features (movement quality, temporal regularity, heart rate dynamics) can predict mobility outcomes, with model choice tailored to the outcome type and the data’s temporal resolution. Integrating multimodal digital biomarkers appears especially promising for capturing nuanced aspects of functional status.

Regarding the types of sensors used in the wearable devices, accelerometry was universal, but inclusion of additional signals (heart rate, heart rate variability, temperature) improved accuracy. Contextual and environmental variables, including weather, moon phase, and financial market indicators, were among the top predictors of PROMIS Pain Interference (AUC 0.92) when combined with wearable features [48]. Similarly, greater accuracy was reported using smartphone features alone (86%) compared to Fitbit features alone (78%), suggesting that contextual or behavioural digital traces may capture hidden aspects of wellbeing not reflected in physiological data [36]. Heart rate and heart rate variability were particularly informative for fatigue, sleep, and cardiovascular outcomes, while multimodal fusion of motion and physiological data generally outperformed single-sensor inputs. Comparative synthesis shows that disease-specific outcomes with clear physiological correlates, such as inflammatory flares or treatment response, achieved the highest accuracies (≥90%), whereas subjective states like fatigue or sleepiness were harder to model (≤65%) [41,42,45,48,55,60,72]. Features linked to gait variability, heart rate variability, and sleep quality emerged as recurring “digital biomarkers” across conditions, supporting their potential as universal indicators of health status.

The reviewed studies demonstrate the growing sophistication of analytical approaches. Classical models such as random forest, support vector machine, and gradient boosting remained dominant and, in most cases, matched or outperformed deep learning approaches, which were rarely implemented and showed no clear advantage on the studied cohorts, with reported model accuracies of 63% [55] and 86% [38]. Temporal modelling, however, appears increasingly important, and accounting for temporal correlations via hidden Markov models resulted in improved accuracy over independent random forest models in two studies (*p* < 0.05) [37,47]. Extensive time-series feature engineering could capture subtle rhythm changes predictive of treatment response, underscoring how simple aggregation into daily averages may obscure meaningful dynamics [62]. Despite this, few studies adopted recurrent neural networks or transformer architectures, representing an opportunity for future work [60,63,73]. The trade-off between predictive power and interpretability remains a key topic. Where simple machine learning models and statistical features offer transparency [39,53], over-engineered feature spaces and deep learning, though potentially more predictive, risk-reducing, and immediate clinical explainability. Careful feature engineering and model design can enhance model explainability by favouring clinically interpretable features, improving alignment between algorithmic predictions and clinical knowledge [74,75,76,77].

Study quality ranged from 12 to 22, with a mean of 18.3, indicating generally moderate to high methodological quality, but not without many common limitations. Most studies involved small, single-centre cohorts, limiting generalisability, and a lack of separate validation datasets is likely to have led to optimistic accuracy estimates [60]. Although several studies reported good to excellent predictive performance, including near-perfect accuracy in some cases, these results should be interpreted with caution. Many of the highest accuracies were observed in studies with very small sample sizes (e.g., *n* ≤ 15) and lacking validation on an external dataset, which substantially increases the risk of overfitting and optimistic performance estimates beyond the cohort on which the model was trained. Models trained and evaluated on the same cohort may capture idiosyncratic patterns specific to the study population alone rather than generalisable disease-related markers. External validation remains limited across this literature, with only two studies identified as employing external validation [35,39] while many relied exclusively on internal validation strategies (e.g., cross-validation, test-train splits), restricting conclusions regarding real-world robustness and highlighting that most current evidence remains proof-of-concept. Furthermore, most studies opted for binary classification. While binary models achieve higher accuracies than multi-class classification or continuous variable prediction, they can lack clinical interpretability, especially when grouped within a cohort (e.g., top and bottom performers) as opposed to externally anchored, clinically meaningful grouping. In addition, binary classification can fail to capture gradations of change relevant to longitudinal patient care. Gossec et al. (2019) initially attempted multi-class flare categorisation (no flare/short flare/persistent flare) but failed to correctly categorise any of the 190 reported persistent flares (sensitivity, 0.0). The model achieved adequate accuracy only after collapsing to binary classification, emphasising the challenge of modelling nuanced ordinal outcomes from noisy behavioural data with limited sample sizes [41]. Data quality and completeness due to non-compliance and technical issues remain a widely reported limitation of wearable studies, and how studies deal with missing data remains inconsistent [78], ranging from removing incomplete days to gap filling with data imputation or averages. Many studies acknowledge that factors such as mood, stress, illnesses unrelated to the primary condition, weather, and medication were often unaccounted for. These factors can confound the observed relationships between wearable data and PROMs, making it challenging to isolate the specific impact of disease states. Finally, a large trade-off exists between predictive power and interpretability due to the “black box” nature of some machine learning models, limiting clinical insights. Only ten studies examined model interpretability and reported feature importance, a critical step for clinical translation [63].

Future research should prioritise larger, multi-centre, longitudinal datasets encompassing diverse demographics and disease states. Such a scale would help reduce model overfitting and enable exploration of advanced temporal models (e.g., recurrent neural networks like long short-term memory architectures) capable of capturing sequential dependencies and trends, as well as anomaly detection capable of identifying health changes potentially indicating disease progression, or the effectiveness of treatment. Interpretability analyses should be standard practice to reveal feature contributions, enhancing explainability and therefore clinical relevance [79,80,81]. Improved data-collection protocols, patient adherence, and higher-resolution sensing (especially for heart rate variability and sleep) are sought to improve model input data quality. Inclusion of demographic, clinical, and contextual variables could enhance model accuracy through personalised predictions and inclusion of confounding factors. Here, integration with electronic health records could further facilitate clinical translation [63]. Finally, for the transition of these predictive models from research proofs-of-concept to clinical tools, rigorous external validation in independent cohorts is necessary, in addition to developing user-friendly formats for clinical use and establishing incentive structures for sustainable implementation.

Recommendations for future research towards real-world implementation

- Larger, multi-centre, longitudinal datasets of diverse demographics and disease states to reduce model overfitting and enable the development of advanced temporal models.
- Interpretability analyses as standard practice.
- Focus on data-collection protocols, specifically patient adherence, and high-resolution sensing for heart rate variability and sleep to improve model input data quality.
- Inclusion of demographic, clinical, and contextual variables to enhance accuracy through personalised predictions and inclusion of confounding factors.
- Rigorous external validation in independent cohorts is necessary.

## 5. Conclusions

While the current body of research suggests immense potential of wearables and machine learning for the prediction of PROMs and PBOMs, evidence of prediction effectiveness remains fragmented and largely exploratory. This review highlighted the requirement for standardised pipelines, transparent reporting, and multi-cohort validation, which are essential before widespread clinical uptake. Non-linear models generally outperform linear models in terms of raw predictive accuracy and handling of high-dimensional wearable data, and that overall, physical function-related PROMs and PBOMs were often predicted with at least moderate accuracy, whereas performance was generally lowest for subjective fatigue scores. These systems have the potential to transform outcome assessment, making it continuous and objective while reducing time and resource strain on patients and healthcare providers. Future progress lies in longitudinal modelling, the collection of large datasets, the inclusion of demographic and contextual information as input features, and a balanced focus on both accuracy and interpretability to ensure that predictive insights translate into trustworthy and generalisable clinical tools.

## Figures and Tables

**Table 1:** Database search strategy.

Search Term	Structure
“wearable *”	OR
“activity tracker *”	OR
“IMU”	AND
“predict *”	OR
*estimat *”	OR
“classif *”	AND
“functional test *”	OR
“outcome measure *”	OR
“self report *”	OR
“self-report *”	OR
“patient report *”	OR
“patient-report”	OR
“questionnaire *”	OR
“functional capacity *”	OR
“clinical test *”	OR
“functional mobility”	

* denotes any truncation.

**Table 2:** Details of wearable devices, device placement, and monitoring periods of all included studies. Abbreviations used: Accel, accelerometer; ECG, Electrocardiography; PPG, photoplethysmography; GPS, global positioning system; EDA, electrodermal activity sensor.

Study	Device	Sensors	Location	Monitoring Period
[55]	VitalPatch (VitalConnect, USA)	3-axis accel, single lead ECG	Chest	5 days
[53]	ActiGraph GT1M (Actigraph, USA)	uniaxial accel	Hip	7 days
[43]	Actibelt sensor (Trium Analysis Online GmbH, Germany)	3-axis accel	Waist	At least one continuous week before each monthly survey answer (for up to 6 months)
[46]	Fitbit Flex (Fitbit, USA)	3-axis accel	Wrist	4 weeks before surgery and 8 weeks postoperatively
	Mio Activity Tracker (Mio Technology, Taiwan)	3-axis accel, PPG	Wrist	
	Lumo Run (Lumo Bodytech, USA)	accel, gyroscope, magnetometer	Waist	
[41]	Withings Activité Pop watch (Withings, France)	3-axis accel	Wrist	3 months
[40]	Fitbit Inspire 2 (Fitbit, USA)	3-axis accel, PPG	Wrist	3 weeks pre-operative
[54]	Apple^®^ Watch Series 3 (Apple Inc., USA)	3-axis accel, barometer, gyroscope, PPG, GPS,	Wrist	6 months
[45]	McRoberts Dynaport device (McRoberts, The Netherlands)	3-axis accelerometer, barometer, magnetometer, temperature sensor	Lower back	2 × 5 consecutive days
[36]	Fitbit Charge HR (Fitbit, USA)	3-axis accel, barometer, PPG	Wrist	4 weeks
[37]	Fitbit Charge 2 (Fitbit, USA)	3-axis accel, barometer, PPG	Wrist	12 weeks
[35]	Not specified	accel, barometer, EDA, PPG, temperature sensor	Wrist	6 h
[48]	Apple^®^ Watch Series 3 (Apple Inc., USA)	3-axis accel, barometer, gyroscope, PPG, GPS	Wrist	6 months
[44]	Not specified	3-axis accel	Wrist	4 days
[47]	Fitbit Versa 2 (Fitbit, USA)	3-axis accel, barometer, PPG	Wrist	12 weeks
[39]	Senior mobility monitor (Philips Research, The Netherlands)	3-axis accel, barometer	Lanyard on chest	12 weeks
[57]	Actigraph GT3X+ (Actigraph, USA)	3-axis accel	Wrist	5 days
[42]	AVIVO™ mobile patient management system (Medtronic Inc., Ireland)	3-axis accel, ECG, temperature sensor	Torso	5 × 7 days over 48 weeks
[38]	Biofourmis Everion (Biofourmis, Singapore)	3-axis accelerometer, gyroscope, PPG, EDA, temperature sensor	Upper Arm	30 days

**Table 3:** Within the study model comparisons.

Model	[43]	[40]	[45]	[37]	[35]	[48]	[44]	[47]	[57]
AdaBoost				X					X
CatBoost						X			
Decision Tree					X				
ExtremeGradientBoost		X			X				
GradientBoost				X	X				X *
Hidden Markov Model				X *				X *	
K Nearest Neighbors		X	X			X			X
Naïve Bayes Classifier			X						X
Neural Network					X		X *		
Regression (lasso)					X				
Regression (linear)					X		X		
Regression (logistic)		X *		X	X	X			X *
Regression (ridge)		X *		X	X				
Random Forest	X *	X	X *	X	X *	X *		X	X
Support Vector Machine	X	X	X *		X	X			X

* denotes best performing model(s).

**Table 4:** Study characteristics and results for quality-of-life prediction. Abbreviations used SD, standard deviation; PROMIS, Patient-Reported Outcomes Measurement Information System; HMM, Hidden Markov Model; AL, activity levels; HR, heart rate; MR, metabolic rate; AUC, area under the curve; RF, random forest; CH, clinical history; GBoost, gradient boost; AHC, agglomerative hierarchical clustering; ADL, activities of daily living; ACC, accuracy; MSKC, musculoskeletal conditions; TJA, total joint arthroscopy.

Outcome Measure	Study	Population *n*, Condition	Quality Score	Classification	Best Model	Features	Result Metric	Result (±SD)
PROMIS physical function	[37]	182, Stable ischemic heart disease	17	Binary: external	HMM	AL, HR, MR, steps	AUC	0.79 (0.02)
	[48]	15, chronic pain	21	Binary: external	RF	AL, CH, HR, steps, weather, moon phase, stock market	AUC	0.78 (0.17)
	[47]	219, rheumatoid arthritis	21	Binary: external	HMM	AL, HR, sleep, steps	AUC	0.75 (0.01)
				Multi-class: external	HMM	AL, HR, sleep, steps	Pearson correlation coefficient	0.48 (0.02)
	[57]	21, chronic pain	18	Multi(3)-class: external	GBoost	AL, rest activity rhythmic features, sleep, steps	AUC	0.56
PROMIS pain interference	[40]	62, Lumbar spine surgery	21	Binary: change	Ridge Regression	HR, sleep, steps	AUC	0.62 (0.04)
	[48]	15, chronic pain	21	Binary: external	RF	AL, CH, HR, steps, weather, moon phase, stock market	AUC	0.92 (0.03)
	[47]	219, rheumatoid arthritis	21	Binary: external	HMM	AL, HR, sleep, steps	AUC	0.66 (0.01)
				Multi-class: external	HMM	AL, HR, sleep, steps	Pearson correlation coefficient	0.33 (0.02)
	[57]	21, chronic pain	18	Multi(3)-class: external	Logistic Regression	AL, rest activity rhythmic features, sleep, steps	AUC	0.62
PROMIS fatigue	[37]	182, Stable ischemic heart disease	17	Binary: external	HMM	AL, HR, MR, steps	AUC	0.65 (0.03)
	[48]	15, chronic pain	21	Binary: external	RF	AL, CH, HR, steps, weather, moon phase, stock market	AUC	0.89 (0.04)
	[47]	219, rheumatoid arthritis	21	Binary: external	HMM	AL, HR, sleep, steps	AUC	0.70 (0.01)
				Multi-class: external	HMM	AL, HR, sleep, steps	Pearson correlation coefficient	0.38 (0.03)
	[42]	10, Barth syndrome	20	Binary: within	AHC	AL, HR, RR	ACC	0.87
				Binary: change	AHC	AL, HR, RR	ACC	1.00
PROMIS sleep disturbance	[37]	182, Stable ischemic heart disease	17	Binary: external	HMM	AL, HR, MR, steps	AUC	0.66 (0.05)
	[48]	15, chronic pain	21	Binary: external	RF	AL, CH, HR, steps, weather, moon phase, stock market	AUC	0.88 (0.10)
	[47]	219, rheumatoid arthritis	21	Binary: external	HMM	AL, HR sleep, steps	AUC	0.65 (0.02)
				Multi-class: external	HMM	AL, HR, sleep, steps	Pearson correlation coefficient	0.20 (0.04)
PROMIS anxiety	[37]	182, Stable ischemic heart disease	17	Binary: external	HMM	AL, HR, MR, steps	AUC	0.61 (0.04)
	[48]	15, chronic pain	21	Binary: external	RF	AL, CH, HR, steps, weather, moon phase, stock market	AUC	0.91 (0.03)
PROMIS depression	[37]	182, Stable ischemic heart disease	17	Binary: external	HMM	AL, HR, MR, steps	AUC	0.59 (0.02)
	[48]	15, chronic pain	21	Binary: external	RF	AL, CH, HR, steps, weather, moon phase, stock market	AUC	0.89 (0.03)
PROMIS social roles	[48]	15, chronic pain	21	Binary: external	RF	AL, CH, HR, steps, weather, moon phase, stock market	AUC	0.88 (0.01)
	[47]	219, rheumatoid arthritis	21	Binary: external	HMM	AL, HR, sleep, steps	AUC	0.67 (0.02)
				Multi-class: external	HMM	AL, HR, sleep, steps	Pearson correlation coefficient	0.32 (0.03)
PROMIS global physical health	[37]	182, Stable ischemic heart disease	17	Binary: external	HMM	AL, HR, MR, steps	AUC	0.76 (0.02)
PROMIS global physical health	[37]	182, Stable ischemic heart disease	17	Binary: external	HMM	AL, HR, MR, steps	AUC	0.61 (0.02)
PROMIS pain intensity	[57]	21, chronic pain	18	3-class: external	Logistic Regression	Rest activity rhythmic features	AUC	0.67
Short Form-36 (mobility and ADL)	[43]	165, Chronic and degenerating MSKC	16	Binary and 3-class: external	RF	Accelerometry, AL, steps	ACC	0.73
Veterans RAND 12-item Health Survey (VR-12)	[46]	22, TJA (hip and knee)	16	3-class: within	K-means	AL, gait analysis, HR, demographics, CH, preoperative AL, sleep	F statistic	17.37

**Table 5:** Study characteristics and results for disease-specific outcomes prediction. Abbreviations used SD, standard deviation; NDD, neurodegenerative disease; IMID, immune-mediated inflammatory diseases; CNN, convolutional neural network; RR, respiratory rate; ACC, accuracy; SVM, support vector machine; AL, activity levels; NBC, Naive Bayes classifiers; NN, neural network; MAE, mean absolute error; HR, heart rate; AUC, area under curve; SPO2, Peripheral Oxygen Saturation; NRS, numerical rating scale; RF, random forest; CH, clinical history; ODI, Oswestry disability index; PHQ-9, Patient Health Questionnaire-9; PGIC, patient global impression of change; PCS, pain catastrophizing scale; AHC, agglomerative hierarchical clustering.

Study	Population *n*, Condition	Quality Score	Best Model	Features	Classification	Outcome Measure	Result Metric	Result (SD)
[55]	82, NDD, and IMID	17	CNN	RR	Binary: external	Karolinska sleepiness scale	ACC	0.63
[45]	72, NDD, and IMID	20	SVM	AL, gait analysis	Binary: within	Fatigue (physical)	ACC	0.57
						Fatigue (mental)	ACC	0.56
[41]	155, Rheumatoid arthritis or Axial spondylarthritis	21	NBC	Steps	Binary: external	Symptom flare	Cohen’s kappa	0.90
[44]	6, Parkinson’s	14	NN	Accelerometry	3-class: external	Tremor score	MAE	~0.30–0.65
[40]	62, Lumbar spine surgery	21	Logistic Regression	HR, sleep, steps	Binary: change	Quality of recovery-15	AUC	0.69 (0.03)
[38]	55, Knee surgery	15	CNN	AL, blood pulse wave, HR, SPO2, perfusion index, RR, skin temperature	Binary: external	NRS	ACC	0.86
					4-class: external	NRS	ACC	0.80
[54]	15, Chronic pain	22	RF	AL, HR, steps, demographics, CH	3-class: external	NRS	ACC	0.78 (0.06)
						PGIC	ACC	0.75 (0.73)
[48]	15, chronic pain	21	RF	AL, CH, HR, steps, weather, moon phase, stock market	3-class: external	NRS	ACC	0.77 (0.01)
						ODI	ACC	0.70 (0.04)
					Binary: external	PGIC	ACC	0.83 (0.06)
						PCS	ACC	0.84 (0.02)
						PHQ-9	ACC	0.85 (0.03)
[35]	7, Cancer	12	RF	HR, skin temperature	Binary: external	Fatigue	ACC	0.68
[36]	14, Cancer (chemotherapy)	18	RF	AL, sleep, steps	3-class: external	MD Anderson symptom inventory	ACC	0.78
[42]	10, Barth syndrome	20	AHC	AL, HR, RR	Binary: within	Barth syndrome symptom assessment	ACC	0.60
					Binary: change	Barth syndrome symptom assessment	ACC	1.00

**Table 6:** Study characteristics and results for performance-based outcome measure prediction. Abbreviations used: SD, standard deviation; AL, activity levels; AUC, area under the curve; CH, clinical history; AHC, agglomerative hierarchical clustering; HR, heart rate; RR, respiratory rate; ACC, accuracy.

Study	Population *n*, Condition	Quality Score	Best Model	Features	Outcome Measure	Classification	Result Metric	Result (SD)
[39]	254, Total hip arthroplasty	22	Regularised linear model	AL, gait analysis, features derived from chair rise events	Timed-up-and-go	Continuous	Spearman correlation coefficient	0.67
						Binary: external	AUC	0.89
[53]	2001, Knee Osteoarthritis	18	Generalised Additive Model	AL, CH, demographics	400 m walk test	3-class: within	Goodman-Kruskal Gamma	0.62 (0.1)
					20 m walk test	3-class: within	Goodman-Kruskal Gamma	0.53 (0.07)
					5 x sit-to-stand	3-class: within	Goodman-Kruskal Gamma	0.51 (0.04)
[42]	10, Barth syndrome	20	AHC	AL, HR, RR	6 min walk test	Binary: within	ACC	0.93
						Binary: change	ACC	1.00
					Handgrip	Binary: within	ACC	0.60
						Binary: change	ACC	1.00
					5 x sit-to-stand	Binary: within	ACC	0.73
						Binary: change	ACC	1.00
					Balance	Binary: within	ACC	0.80
						Binary: change	ACC	1.00

**Table 7:** Study characteristics and results for predicting mobility outcomes. Abbreviations used: SD, standard deviation; HMM, hidden Markov model; AL, activity levels; HR, heart rate; MR, metabolic rate; PROMIS, Patient-Reported Outcomes Measurement Information System; AUC, area under curve; RF, random forest; CH, clinical history; GBoost, gradient boost; MSKC, musculoskeletal condition; ACC, accuracy; AHC, agglomerative hierarchical clustering.

Study	Population *n*, Condition	Quality Score	Best Model	Features	Outcome Measure	Classification	Result Metric	Result (SD)
[37]	182, Stable ischemic heart disease	17	HMM	AL, HR, MR, steps	PROMIS physical function	Binary: external	AUC	0.79 (0.02)
[47]	219, rheumatoid arthritis	21	HMM	AL, HR, sleep, steps	PROMIS physical function	Binary: external	AUC	0.75 (0.01)
						Multi-class: external	Pearson correlation coefficient	0.48 (0.02)
[48]	15, chronic pain	21	RF	AL, CH, HR, steps, weather, moon phase, stock market	PROMIS physical function	Binary: external	AUC	0.78 (0.17)
[57]	21, chronic pain	18	GBoost	AL, rest activity rhythmic features, sleep, steps	PROMIS physical function	3-class: external	AUC	0.56
[43]	165, Chronic and degenerating MSKC	16	RF	Accelerometry, AL, steps	Short Form-36 (mobility/activities)	Binary & 3-class: external	ACC	0.73
[39]	254, Total hip arthroplasty	22	Regularised linear model	AL, gait analysis, features derived from chair rise events	Timed-up-and-go	Continuous	Spearman correlation coefficient	0.67
						Binary: external	AUC	0.89
[53]	2001, Knee Osteoarthritis	18	Generalised Additive Model	AL, CH, demographics	400 m walk test	3-class: within	Goodman-Kruskal Gamma	0.62 (0.1)
					20 m walk test	3-class: within	Goodman-Kruskal Gamma	0.53 (0.07)
					5 x sit-to-stand	3-class: within	Goodman-Kruskal Gamma	0.51 (0.04)
[42]	10, Barth syndrome	20	AHC	AL, HR, RR	6 min walk test	Binary: within	ACC	0.93
						Binary: change	ACC	1.00
					Handgrip	Binary: within	ACC	0.60
						Binary: change	ACC	1.00
					5 x sit-to-stand	Binary: within	ACC	0.73
						Binary: change	ACC	1.00
					Balance	Binary: within	ACC	0.80
						Binary: change	ACC	1.00

**Table 8:** Study characteristics and results for the prediction of pain. Abbreviations used SD, standard deviation; AL, activity levels; PROMIS, Patient-Reported Outcomes Measurement Information System; RF, random forest; CH, clinical history; HR, heart rate; NRS, numerical rating scale; AUC, area under curve; CNN, convolutional neural network; SPO2, Peripheral Oxygen Saturation; RR, respiratory rate; ACC, accuracy; HMM, Hidden Markov Model.

Study	Population *n*, Condition	Quality Score	Best Model	Features	Classification	Outcome Measure	Result Metric	Result (SD)
[57]	21, Chronic pain	18	Logistic regression	AL, rest activity rhythmic features, sleep, steps	3-class: external	PROMIS pain interference	F1	0.59
						PROMIS pain intensity	F1	0.60
[48]	15, Chronic pain	21	RF	AL, CH, HR, steps, weather, moon phase, stock market	Binary: external	PROMIS pain interference	F1	0.85 (0.06)
					3-class: external	NRS	F1	0.77 (0.01)
[54]	15, Chronic pain	22	RF	AL, HR, steps, demographics, CH	3-class: external	NRS	F1	0.73 (0.08)
[40]	62, Lumbar spine surgery	21	Ridge Regression	HR, sleep, steps	Binary: change	PROMIS pain interference	AUC	0.62 (0.04)
[38]	55, Knee surgery	15	CNN	AL, blood pulse wave, HR, SPO2, perfusion index, RR, skin temperature	Binary: external	NRS	ACC	0.86
					4-class: external	NRS	ACC	0.80
[47]	219, rheumatoid arthritis	21	HMM	AL, HR, sleep, steps	Binary: external	PROMIS pain interference	AUC	0.66 (0.01)
					Multi-class: external	PROMIS pain interference	Pearson correlation coefficient	0.33 (0.24)

## Data Availability

The data presented in this study are available on request from the corresponding author.

## Abbreviations

The following abbreviations are used in this manuscript:

PROMs	Patient-reported outcome measures
PBOMs	Performance-based outcome measures
PROMIS	Patient-Reported Outcomes Measurement Information System
TUG	Timed up-and-go
6MWT	Six-minute walk test
ECG	Electrocardiography
PROSPERO	International Prospective Register of Systematic Reviews
PRISMA	Preferred Reporting Items for Systematic Reviews and Meta-Analyses
ICD-11	International Classification of disease-11
PPG	Photoplethysmography
AUC	Area Under Curve
BMI	Body mass index
SHAP	SHapley additive exPlanations
